# Development and evaluation of a candidate reference measurement procedure for detecting 17α-hydroxyprogesterone in dried blood spots using isotope dilution liquid chromatography tandem mass spectrometry

**DOI:** 10.1007/s00216-024-05411-9

**Published:** 2024-06-29

**Authors:** Ziyun He, Haibing Dai, Jian Shen, Yanjie Huang, Jinsong Liu, Renqing Yan, Feng Zhang, Shengkai Yan

**Affiliations:** 1 Department of Laboratory Medicine, Affiliated Hospital of Zunyi Medical University, Zunyi, 563003 China; 2 College of Laboratory Medicine, Zunyi Medical University, Zunyi, 563000 China; 3 Guangzhou Fenghua Biotech Co., Ltd., Guangzhou, 510730 China; 4 Guangdong Provincial Institute of Metrology, South China National Centre of Metrology, Guangzhou, 510405 China

**Keywords:** Congenital adrenal hyperplasia, 17α-Hydroxyprogesterone, Dried blood spots, Liquid chromatography tandem mass spectrometry, Newborn screening

## Abstract

17α-Hydroxyprogesterone (17α-OHP) quantification in dried blood spots (DBS) is essential for newborn screening for congenital adrenal hyperplasia (CAH), which is challenging due to its low physiological concentration. The high false-positive rates of immunoassays necessitate the development of more accurate methods. Liquid chromatography tandem mass spectrometry (LC-MS/MS) offers increased specificity and sensitivity, yet standardized procedures for 17α-OHP measurement are required for clinical application. A candidate reference measurement procedure (cRMP) using isotope dilution LC-MS/MS was developed for 17α-OHP quantification in DBS. By utilizing stable isotope-labeled D8-17α-OHP as an internal standard, the cRMP was optimized, covering sample preparation, calibration, and LC-MS/MS analysis. The method performance was validated across several parameters, including precision, accuracy, specificity, detection limits, and matrix effects. Clinical applicability was further assessed through the establishment of reference intervals for healthy newborns. The developed cRMP exhibited a linear range of 1.00 to 80.00 ng/mL for 17α-OHP, with detection and quantification limits of 0.14 ng/mL and 0.52 ng/mL, respectively. Inter- and intraday precision demonstrated coefficients of variation within 1.27 to 5.69%. The recovery rates and matrix effects were well within acceptable limits, ensuring method reliability. Clinical application showed distinct reference intervals for healthy newborns that were unaffected by sex but influenced by weight and gestational age. This method significantly enhances CAH diagnostic accuracy in newborns, providing a valuable tool for clinical laboratories and improving newborn screening program standardization and traceability.

**Supplementary Information:**

The online version contains supplementary material available at 10.1007/s00216-024-05411-9.

## Introduction

17α-Hydroxyprogesterone (17α-OHP), an endogenous steroid hormone, plays a crucial role in the biosynthesis of glucocorticoids and sex steroid hormones. The enzyme 21-hydroxylase catalyzes its conversion to 11-deoxycortisol, a precursor in cortisol synthesis. This hormonal cascade is essential, as disruptions can lead to adrenocortical hyperplasia due to decreased cortisol levels and consequent negative feedback mechanisms [1]. During pregnancy, the fetus, placenta, and adrenal glands markedly produce 17α-OHP, leading to elevated post-natal blood levels in normal newborns, which typically return to baseline within 24 h [2]. As an intermediate in the synthesis of adrenocorticotropic hormone, the conversion of 17α-OHP to estrogens and androgens is pivotal for maintaining reproductive system balance and sex hormone equilibrium.

The diagnosis of congenital adrenal hyperplasia (CAH), an autosomal recessive disorder characterized by enzyme defects in adrenocorticotropic hormone synthesis, in newborns primarily depends on measuring 17α-OHP levels [3]. The approximate incidence of CAH is 1/16,000, and its potential for grave outcomes, such as hyperandrogenemia and salt-loss crisis, underscores the necessity for accurate diagnostic methods [4].

Traditionally, immunoassays have been employed for initial CAH screening via 17α-OHP level detection in dried blood spots (DBS) or serum. However, immunoassays often yield falsely elevated 17α-OHP levels in preterm infants and those with non-CAH diseases [5]. As immunoassays based on antigen-antibody responses are prone to false-positive results, secondary screening of these children is required [6]. The most common secondary screening method for detecting 17α-OHP is liquid chromatography tandem mass spectrometry (LC-MS/MS) [7]. The adoption of LC-MS/MS as a secondary screening tool, as advocated by Kao et al. [8], represents a paradigm shift toward more accurate and reliable diagnosis of adrenocortical dysfunctions, reducing false positives and enhancing specificity over traditional immunoassays [9–11].

Several studies have been conducted to refine LC-MS/MS techniques for steroid hormone detection, enhancing the diagnostic accuracy of newborn CAH screening. Zhan developed an LC-MS/MS method for the quantification of 17α-OHP in serum, enhancing the accuracy of newborn CAH screening [12]. Choi developed and validated a method for the simultaneous quantification of nine steroid hormones in DBS samples using LC-MS/MS [13]. Reference intervals for these hormones were also established, contributing to the clinical applicability of the method. Qasrawi and Sadrzadeh advanced this field by developing an LC-MS/MS assay capable of quantifying seven steroids from DBS to diagnose CAH [14]. They underscored the superior analytical performance of the assay over traditional immunoassays, validating the method according to Clinical and Laboratory Standards Institute (CLSI) guidelines. These advancements include the development of assays, highlighting the potential of LC-MS/MS for comprehensive and precise CAH diagnostics.

A candidate reference measurement procedure (cRMP) was developed to provide a highly accurate and reliable method for measuring a specific analyte in biological samples, which can then be used as a benchmark or standard against which other measurement procedures can be compared or calibrated [15]. The primary purpose of a cRMP is to ensure that measurements of specific analytes, such as 17α-OHP in this context, are consistent, accurate, and traceable to international standards. This consistency is crucial for accurately diagnosing conditions such as CAH across different laboratories and testing platforms [16].

We studied the use of a cRMP for detecting 17α-OHP in DBS via isotope dilution liquid chromatography tandem mass spectrometry (ID-LC-MS/MS). The main advantage of this method lies in its use of stable isotope labeling as an internal standard (IS), which significantly enhances the measurement reliability. By providing a robust framework for the accurate quantification of 17α-OHP in DBS, cRMP represents a significant advancement in the field, promising improved diagnostic confidence and traceability in clinical settings.

## Experimental

### Reagents and materials

Whole-blood and DBS samples were obtained from healthy volunteers at the First Affiliated Hospital of Zunyi Medical College between January and December 2023. The data were analyzed in a completely anonymous manner, thus eliminating the need for informed consent. The study was approved by the Ethics Committee of Zunyi Medical University. Steroid-free serum, confirmed to be free of infectious disease hazards, was obtained from Beijing Hua Xinhang Biotechnology Co., Ltd. (CAT No. NCSHZ500-S).

Analytical-grade methanol and acetonitrile were acquired from Merck (Darmstadt, Germany, CAS No.67-56-1 and 75-05-8, respectively), while formic acid was obtained from Sigma (St. Louis, MO, USA, CAS No. 540-69-2). Additionally, ammonium formate, acetate, and fluoride were purchased from Shanghai Aladdin Biochemical Science and Technology Company Limited (Shanghai, China). The 17α-OHP certified reference material (CRM) was obtained from the National Institute of Metrology (Beijing, China, Certificate No. GBW 09220, CAS No. 68-96-2), and the D8-17α-OHP isotope was procured from BePure (Beijing, China, CAS No. 850023-80-2). Water for preparing standard solutions, sample solutions, and the mobile phase was purified using a Millipore Alpha-Q water purification system (Millipore, Billerica, MA, USA).

### Calibration procedure for the analysis of DBS

The calibration process involved accurate weight of 17α-OHP CRM using a high-precision analytical balance (Sartorius, Germany); the CRM was then dissolved in methanol to create a high-concentration stock solution in the mg/mL range. This mixture was subsequently diluted 10,000-fold with methanol to obtain a low-concentration variant. Blood samples from healthy adult volunteers were collected in heparin anticoagulation tubes, centrifuged to separate plasma, and extensively washed with saline to eliminate plasma residue. The resultant cells were combined with steroid-free serum at a 55:45 volume ratio to produce steroid-free whole blood. The low-concentration stock solution was first added to containers and then evaporated under a nitrogen stream, and subsequently, steroid-free whole blood was added, resulting in the preparation of a spiked whole-blood matrix. The spiked whole blood was gently agitated for 30 min and allowed to stabilize at room temperature for at least 1 h.

Calibrators were prepared by pipetting 50 μL of spiked whole-blood matrix onto Whatman 903 filter paper, drying overnight at room temperature, storing in foil pouches at − 20 °C, and utilizing the matrix promptly upon opening. The DBS calibrator concentrations were set at 1, 10, 30, 50, 70, and 80 ng/mL. Quality control (QC) samples for DBS 17α-OHP were obtained from a time-resolved fluorescence immunoassay (TRFIA) kit (Guangzhou Fenghua Biotech Co., Ltd., China). The isotope D8-17α-OHP was precisely weighed, dissolved in methanol, and diluted to a 10 µg/mL stock solution, which was stored at − 20 °C until use. A working solution of the IS at a concentration of 2 ng/mL was prepared using a methanol/acetonitrile mixture (1:1, v/v) on the day of analysis.

### Sample preparation

Two 3.2 mm diameter blood spots were extracted from the DBS calibrators and QC samples using a puncher and placed into a 96-well microtiter plate (Thermo Fisher, USA). Subsequently, 100 μL of a working solution with a D8-17α-OHP concentration of 2 ng/mL was added to each well. The plate was then sealed and centrifuged at 2000 rpm for 10 s, followed by agitation at 750 rpm for 50 min at a controlled temperature of 25 °C. After centrifugation, the supernatant was carefully transferred to a 96-well polypropylene plate with a pointed bottom and evaporated under nitrogen at 50 °C. It was then reconstituted in 70 μL of 50% methanol in water (v/v,1:1), agitated at room temperature for 5 min at 750 rpm or allowed to stand for more than 10 min to ensure complete re-dissolution before analysis.

### LC-MS/MS analysis

LC-MS/MS analysis was conducted using an Acquity UPLC system coupled to a Xevo TQS mass spectrometer (Waters, USA). Chromatographic separation was achieved on a Waters Acquity BEH C18 column (1.7 μm, 2.1 mm × 50 mm) protected by a guard column (2.1 mm × 5 mm), with the column oven and sample tray temperatures maintained at 40 °C and 10 °C, respectively. The mobile phases comprised water with 0.2 mmol/L NH_4_F (MPA) and methanol (MPB). The gradient elution commenced at 45% MPB, increased linearly to 80% MPB over 2 min, then to 98% MPB within the next minute, maintained for 1.5 min, quickly reverted to 45% MPB, and stabilized for 0.5 min. The flow rate was 0.3 mL/min, with a total runtime of 5 min and an injection volume of 5 μL. Measurements were performed in positive electrospray ion mode using argon as the collision gas. The optimized MS/MS transitions for the analytes were 331.2 > 97.2 and 331.2 > 109.1 for 17α-OHP and 339.3 > 100.2 and 339.3 > 113.2 for D8-17α-OHP.

## Results

### Optimization of sample preparation and method conditions

Methanol, acetonitrile, isopropanol, and water were evaluated as extraction solvents for the analysis of DBS 17α-OHP, and the temperature and duration of the extraction process were also meticulously optimized. The mobile phase was optimized utilizing formic acid, ammonium formate, ammonium acetate, and ammonium fluoride, and various chromatographic columns were evaluated to enhance the chromatographic peak shape and area. A calibration curve was constructed using six calibrators at different concentrations, each analyzed three times in a single day to determine the correlation coefficient (*r*^2^) based on the ratio of 17α-OHP/IS peak areas against the concentration of 17α-OHP. A calibration curve with *r*^2^ > 0.999 was deemed acceptable.

**Fig. 1:**
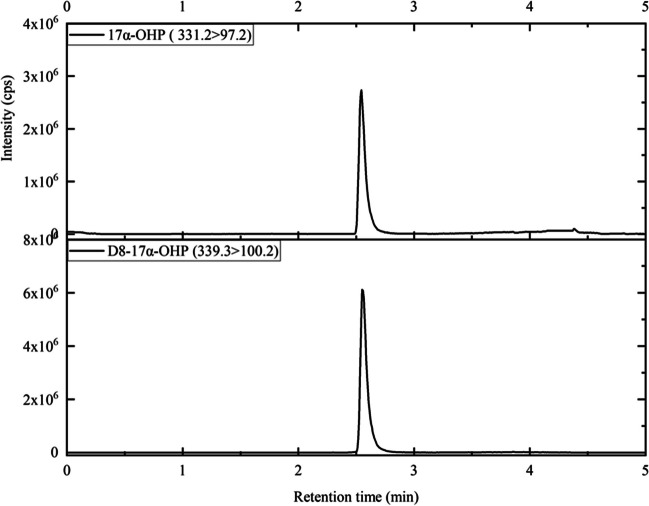
Ion chromatograms of both 17α-OHP and its isotopic analog. The SRM transition for 17α-OHP is 331.2 > 97.2, while the SRM transition for D8-17α-OHP is 339.3 > 100.2

**Fig. 2:**
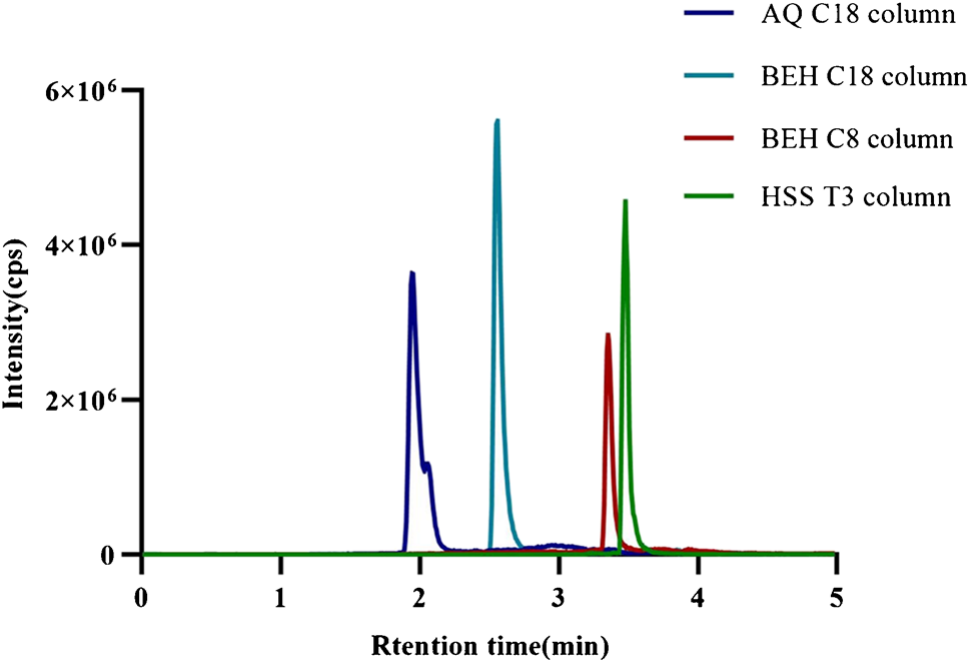
The total ion chromatograms of 17α-OHP were obtained using four distinct columns: an AQ C18 column (2.1 × 50 mm, 2.6 μm, Thermo Fisher, USA), a BEH C18 column (2.1 × 50 mm, 1.7 μm, Waters, USA), a BEH C8 column (2.1 × 100 mm, 2.5 μm, Waters, USA), and an HSS T3 column (2.1 × 100 mm, 1.8 μm, Waters, USA)

A typical chromatogram of the treated DBS sample is presented in Fig. 1, demonstrating effective separation achieved via gradient elution within a concise timeframe of 5 min. The standard curve was constructed by plotting the labeled concentration of the calibrators on the x-axis (X) against the ratio of the actual detected peak area to the peak area of the IS (Y) on the y-axis. The regression equation for the curve is then calculated, with the *r*^2^ value being greater than 0.999, as illustrated in Fig. S1. The ion selection process for 17α-OHP and D8-17α-OHP is shown in Fig. S2. The quantification range for 17α-OHP was established between 1.00 and 80.00 ng/mL. Details of the optimized gradient for the chromatographic mobile phase are provided in Table S1, while the refined parameters for mass spectrometric analysis are outlined in Table 1. After evaluating the four chromatographic columns, a Waters Acquity BEH C18 column was selected because of its superior peak shapes and response intensities, as shown in Fig. 2.

**Table 1:** Optimized MS/MS parameters

MS settings	Parameters
Ionization mode	ESI +
Transitions	MRM
Capillary, kV	1.5
Cone, V	32
Source temperate, °C	150
Desolvation temperature, °C	550
Cone gas flow, L/h	150
Desolvation gas flow, L/h	900
Collision gas flow, mL/min	0.18 (argon)

*ESI* + electrospray ionization positive ion mode, *MRM* multiple reaction monitoring

**Fig. 3:**
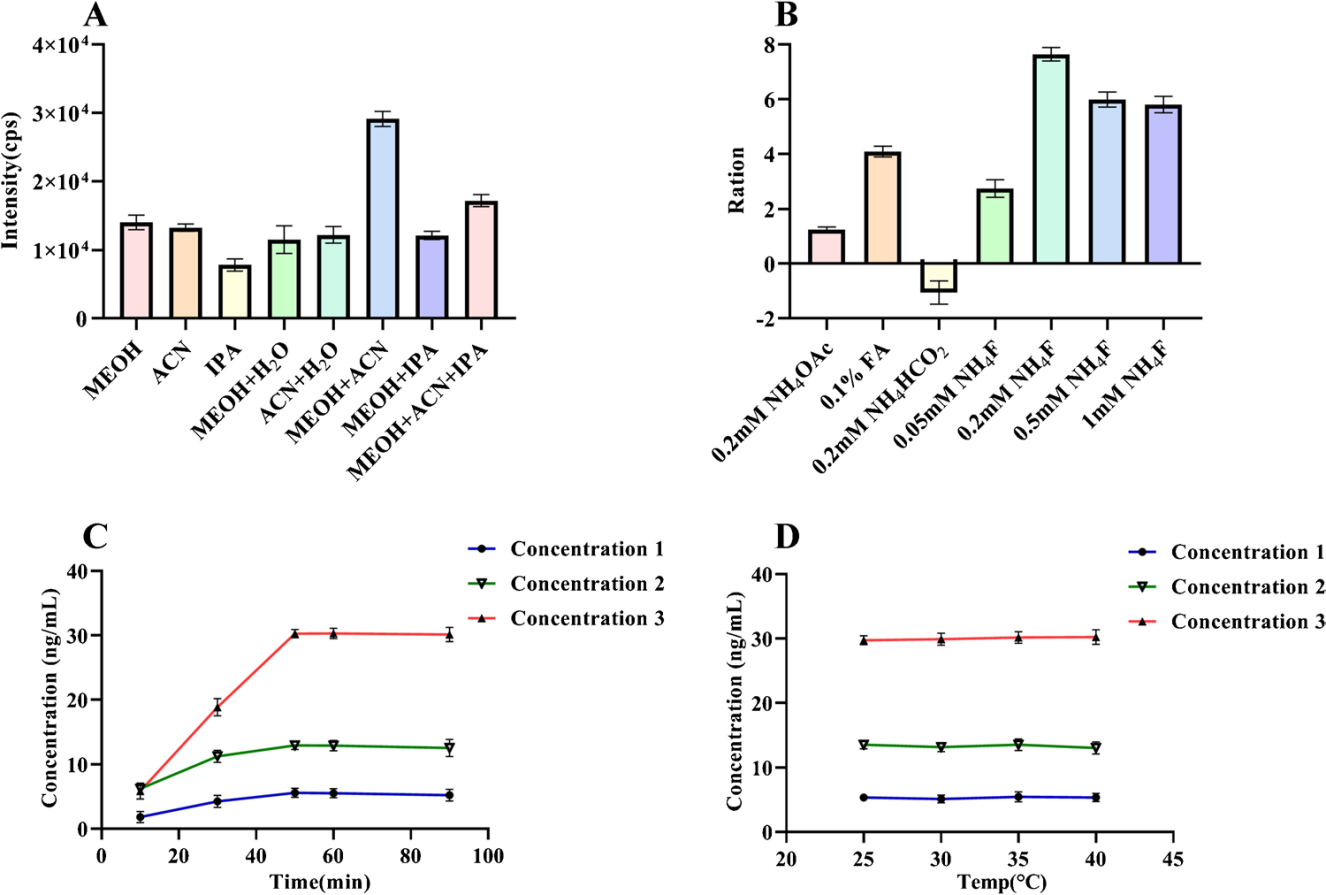
**A** Selection of extraction solution; **B** selection of mobile phase additives; **C** optimization of extraction time; **D** optimization of extraction temperature; MEOH, methanol; ACN, acetonitrile; IPA, isopropanol; NH_4_OAC, ammonium acetate; FA, formic acid; NH_4_F, ammonium fluoride; NH_4_HCO_2_, ammonium formate

In this study, we employed various solvents and additives to optimize the extraction and analysis processes. To identify the optimal extraction solvent, we evaluated the extraction efficiencies of methanol, acetonitrile, and isopropanol both individually and in combination with water. The highest extraction efficiency was achieved using 1:1 methanol/acetonitrile (v/v). Subsequently, the composition of the mobile phase was optimized by comparing the peak area ratios obtained with various additives to those achieved with a methanol-water mobile phase. The addition of 0.2 mmol/L ammonium fluoride (MPA) yielded the most stable and pronounced response. Furthermore, the ideal extraction conditions were established at a temperature of 25 °C and a duration of 50 min. The outcomes of the sample preparation optimization are presented in Fig. 3.

### Precision

The precision was quantified using the coefficient of variation (CV). This approach involved conducting intraday variability assessments by measuring three sets of QC samples five times on the same day, and interday variability was determined by repeating this assessment over five consecutive days. In the validation process, the intraday and interday precision ranged from 1.27 to 5.69% and 1.66 to 5.78%, respectively, as detailed in Table S2.

### Accuracy

Due to the absence of a DBS reference measurement procedures (RMP) or matrix reference materials for 17α-OHP in the Joint Committee for Traceability in Laboratory Medicine database, accuracy was evaluated through spiked recovery experiments. Initially, a base whole-blood matrix containing a specific concentration of 17α-OHP was prepared. The base whole blood was then divided into four portions. Three of these portions were subsequently spiked with different concentrations of 17α-OHP standard solution to create spiked whole-blood samples containing 17α-OHP at low, medium, and high concentrations. Both the base whole-blood and the spiked whole-blood samples were then processed to form DBS. All four types of DBS were tested. Take 5 samples for each concentration, and repeat the measurement three times for each sample within 1 day, and the recovery rate of 17α-OHP in the DBS was calculated. The formula for calculating the recovery rate was the measured value of the spiked DBS minus the measured value of the base DBS divided by the theoretical value of the amount of added 17α-OHP. External quality assurance (EQA) from the National Center for Clinical Laboratories (CAT NO. NCCL-E-09-03-2022) further validated the accuracy of the method. Each EQA sample is tested three times within 1 day.

The recovery rates for 17α-OHP in DBS, as determined by ID-LC-MS/MS, ranged from 91.22 to 105.71%. The CV was maintained at or below 8% as detailed in Table 2. To assess the reliability of the method with real-world samples, EQA samples provided by the NCCL (code NCCL-E-09-03-2022) were utilized. The measured values were compared to the certified reference values, with all outcomes residing within the defined acceptable limits, as shown in Table 3.

**Table 2:** Results of spiked recoveries

Sample name	Measured mean (ng/mL)	Recovery (%)	CV (%)
Sample-L-1	5.03 ± 0.05	101.00	1.04
Sample-L-2	4.75 ± 0.14	91.67	2.99
Sample-L-3	5.05 ± 0.17	101.67	1.39
Sample-L-4	4.92 ± 0.15	97.33	3.00
Sample-L-5	4.92 ± 0.11	97.67	2.29
Sample-M-1	19.08 ± 0.06	94.89	0.31
Sample-M-2	18.42 ± 0.04	91.22	0.22
Sample-M-3	19.41 ± 0.13	96.72	0.68
Sample-M-4	20.52 ± 0.24	102.44	1.11
Sample-M-5	19.26 ± 0.25	95.89	1.29
Sample-H-1	52.66 ± 0.22	105.71	0.41
Sample-H-2	49.46 ± 0.13	98.88	0.27
Sample-H-3	50.95 ± 0.21	101.98	0.41
Sample-H-4	50.38 ± 0.47	100.77	0.94
Sample-H-5	48.61 ± 0.31	97.10	0.63

**Table 3:** Validation of EQA materials

EQA	Target values (ng/mL)	Measured mean (ng/mL)	Deviation (%)
NNCL-2022231	4.32	4.16 ± 0.26	− 3.70
NNCL-2022232	29.96	30.62 ± 0.94	2.20
NNCL-2022233	39.14	37.65 ± 0.78	− 3.81
NNCL-2022234	3.86	3.63 ± 0.25	− 5.96
NNCL-2022235	28.87	29.30 ± 0.53	1.48

Sample-L-1 represents low-concentration sample 1, and so on; Sample-M-1 represents medium-concentration sample 1; Sample-H-1 represents high-concentration sample 1.

### Specificity

Interference tests were conducted with known high-concentration interferents to assess the impact of jaundice, hemolysis, and lipemia on the analyte measurements. Additionally, the influence of endogenous substances such as progesterone (P), testosterone (T), androstenedione (A4), 16α-OHP, 11α-OHP, and 21-deoxycorticosterone (21-DOC) was examined.

**Fig. 4:**
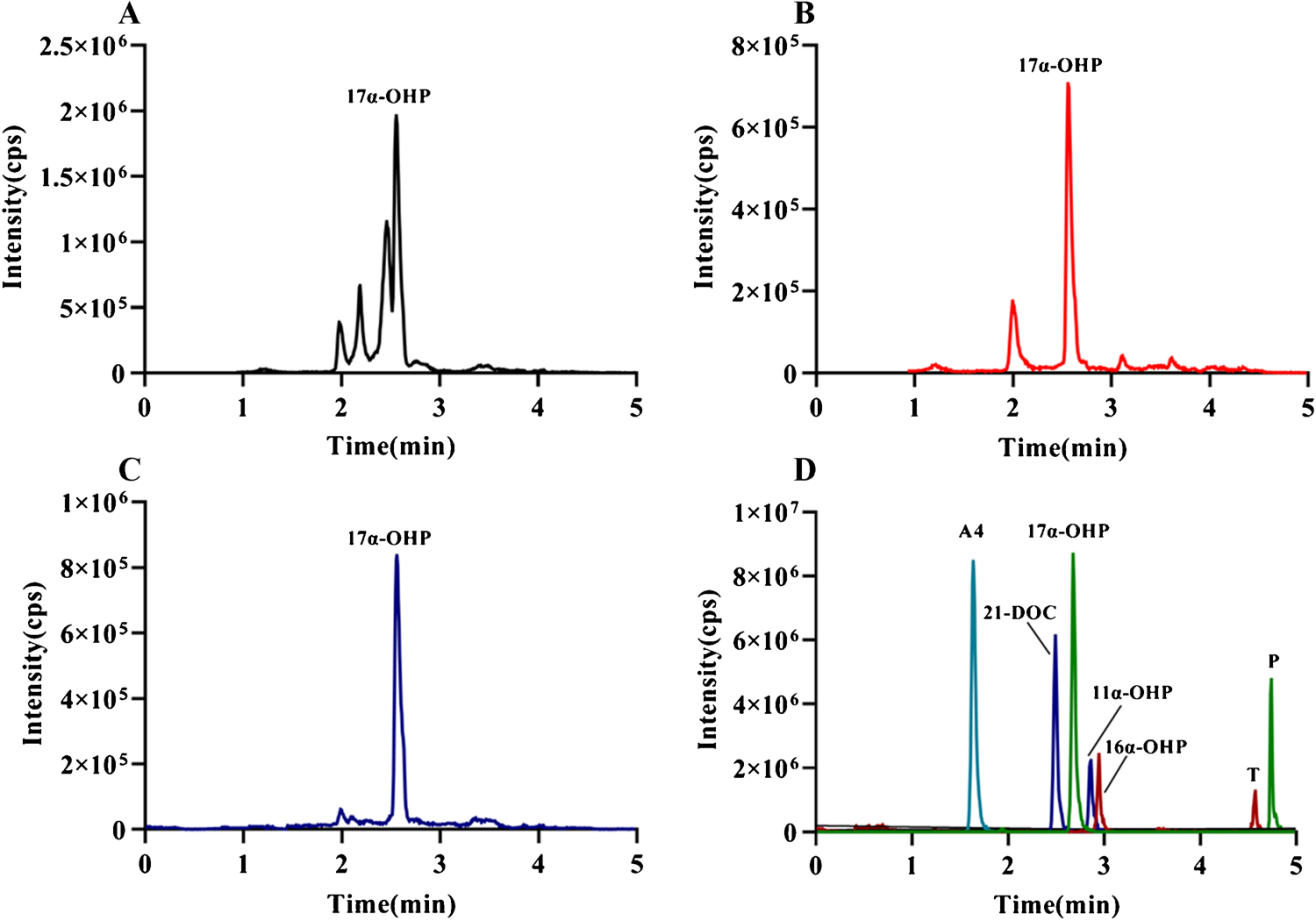
The specificity test total ion chromatograms revealed distinct outcomes for various DBS sample types. **A** Completely hemolyzed samples; **B** high-fat samples; **C** jaundice samples; **D** endogenous hormones and structural analogs in total ion chromatography. The endogenous hormones examined included A4, P, T, 11α-OHP, 16α-OHP, 21-DOC, and 17α-OHP at 10 ng/mL

Validation demonstrated that neither jaundice nor elevated triglyceride levels impacted the quantification of 17α-OHP. However, samples that were completely hemolyzed contained substances that coeluted with 17α-OHP, resulting in merged chromatographic peaks and thereby affecting its quantification. Furthermore, the quantification of 17α-OHP was unaffected by endogenous hormones such as P, A4, and T. Similarly, the quantification of 17α-OHP was not influenced by its structural analogs 11α-OHP, 16α-OHP, or 21-DOC as illustrated in Fig. 4. Despite the close elution of the chromatographic peaks of these structural analogs, they could be completely separated and did not interfere with each other in their respective quantification channels.

### Method comparison and matrix effects

Clinical samples were subjected to LC-MS/MS and TRFIA (Wallac Oy, Finland) analyses. Clinical samples consisted of residual sodium heparin-anticoagulated whole blood, of which five had high levels of 17α-OHP (> 15 ng/mL). We defined high values with reference to Mayo Clinic Laboratories (https://www.mayocliniclabs.com/). All the samples were analyzed simultaneously by two methods. The matrix effect was evaluated using the post-sample addition method. A particular target analyte concentration was spiked into a pretreated blank matrix sample. The spiked treated matrix sample and the same 50% methanol solvent sample concentration were analyzed together.

**Fig. 5:**
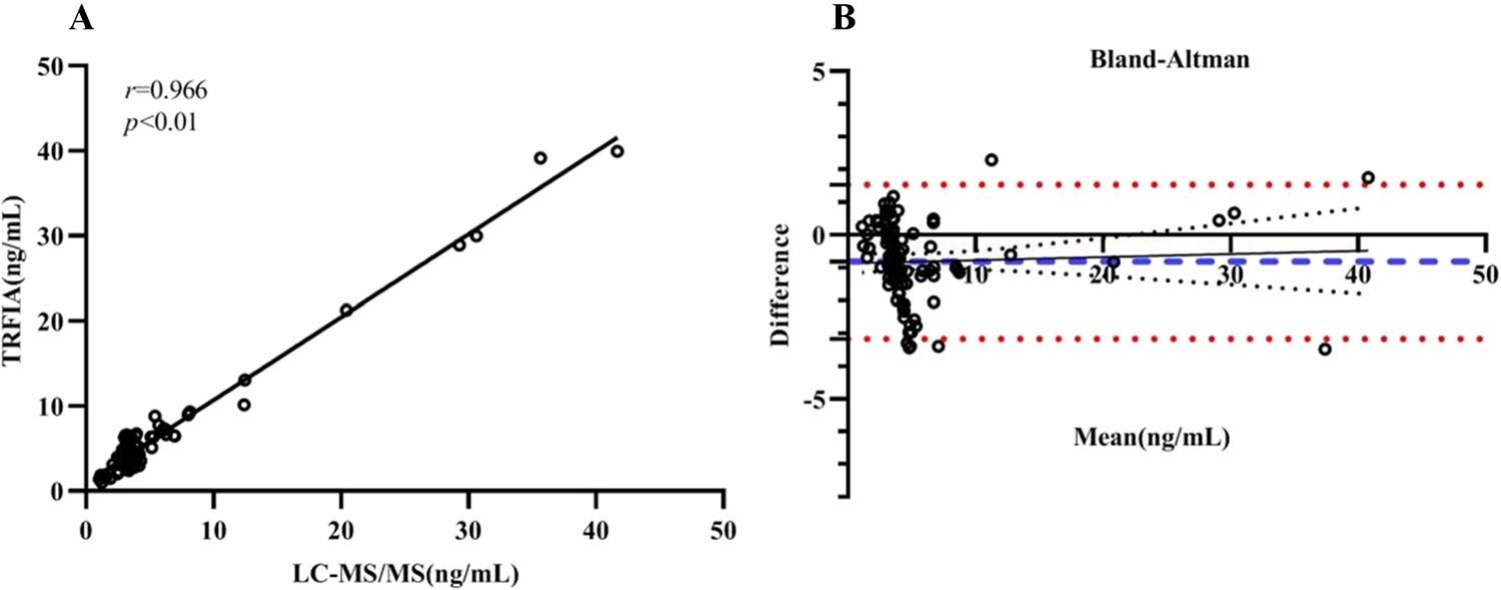
**A** Passing‒Bablok regression (*n* = 98) slope: 0.974 [0.937 to 1.011]; intercept: 0.958 [0.651 to 0.625] (*r* = 0.996, *P* < 0.01). **B** Bland‒Altman plots for LC-MS/MS and TRFIA analysis of clinical samples (*n* = 98). Bias =  − 0.82, 95% limits of agreement =  − 3.17 to 1.53. The red dashed lines represent one standard deviation above and below the mean, while the blue dashed lines indicate the mean bias

Ninety-eight clinical samples were collected for method comparison analysis, employing ID-LC-MS/MS as the comparison method and TRFIA as the evaluation method. In the correlation analysis, the comparison method results are plotted on the x-axis, and the evaluation method results are plotted on the y-axis. The data points were fitted on a scatter plot to determine the correlation between the two methods (Fig. 5A). The results show a poor correlation between the two methods, particularly in high-concentration samples. The Bland‒Altman plot, with the x-axis representing the average measured concentrations and the y-axis showing the differences between the comparison and evaluation methods, was used to determine the distribution of discrepancies between the two measurement approaches, as shown in Fig. 5B. The intermethod comparison test results demonstrated an average bias of − 13.7% between the comparison and evaluation methods. Additionally, a comparison revealed a negative matrix effect in DBS relative to solvent standards, with the calculated matrix effects ranging from − 6.49 to − 9.78%, as detailed in Table S3.

### Carryover

Low-concentration samples were repeatedly injected, and then high- and low-concentration samples were alternately injected. We compared the differences in detection results between low-concentration samples injected consecutively and low-concentration samples injected alternately. No significant carryover contamination was observed in cross-injected samples at high or low concentrations, as shown in Table S4.

### Quantification and detection limits

The method sensitivity was established by serial dilutions of the lowest concentration calibrator, assessing the signal-to-noise ratio (S/N), CV, and deviation from the theoretical value. The lower limit of detection (LOD) was defined as the lowest concentration achieving S/N > 3, CV ≤ 20%, and deviation ≤ 15%. The lower limit of quantification (LOQ) was defined as the lowest concentration achieving S/N > 10, CV ≤ 20%, and deviation ≤ 15%.

Dilution experiments on low-concentration QC samples indicated that 17α-OHP can be accurately quantified in DBS at the third and fourth dilutions (1.40 to 0.70 ng/mL), with no peak signal detected at the seventh dilution (0.08 ng/mL). Consequently, to establish the LOQ and LOD, five samples were prepared within a concentration range of 0.60 to 1.00 ng/mL. The method LOQ was set based on sample 4’s mean value of 0.52 ng/mL, with a CV of 4.73% and a bias of 3.00%. The mean value of sample 2 of 0.14 ng/mL was set as the LOD (Table S5). Moreover, the LOQ and LOD were conditional upon the extraction from two 3 mm DBS using 100 μL of extraction solution and resuspension in 70 μL of solution for a 5 μL injection volume. An increase in the method sensitivity and a corresponding reduction in the LOD could be achieved by increasing the DBS extraction volume, decreasing the resuspension solution volume, or increasing the injection volume. Nevertheless, given the clinical physiological concentration ranges of the biomarkers, the present LOD is sufficient for the quantification of 17α-OHP in DBS.

### Measurement uncertainty assessment

The measurement uncertainty was evaluated according to the ISO Guide to the Expression of Uncertainty in Measurement. Type A uncertainty assessment refers to the uncertainty derived from the results of repeated measurements of the same physical quantity, typically encompassing both systematic and random errors, and the calculation is based on the statistical analysis of a series of repeated measurements, reflecting the consistency and precision of the measurement process.

Type B uncertainty assessment refers to the uncertainty arising from the measuring instruments, equipment, or standards used in the measurement process and typically includes uncertainties related to calibration, uncertainties specified by the manufacturer, and changes in environmental conditions. The specific calculations are detailed below. (1) Calibration uncertainty introduced by the mass spectrometer: As a highly precise analytical instrument, the uncertainty introduced during the calibration of the mass spectrometer, after thorough evaluation, can be considered negligible. (2) Relative standard uncertainty introduced by the purity of the standard substance: According to the certificate of the standard substance, the purity of the 17α-OHP standard was 99.3%, with an expanded uncertainty of 0.2% (*k*_CRM_ = 2). The relative standard uncertainty introduced by the purity of the standard substance was 0.2% / (2 × 99.3%) = 0.10%. (3) Relative standard uncertainty introduced by the use of the analytical balance: The uncertainty of balance weighing stems from the repeatability of weighing and the uncertainty of the balance itself. (4) Relative standard uncertainty introduced by the pipette: The sample extraction process mainly uses a 100 μL single-channel pipette, where errors caused by volume and errors introduced by environmental temperature are calculated separately to assess their introduced relative uncertainty. (5) Relative standard uncertainty introduced by volumetric flasks: The calibration samples were prepared in 100 mL and 10 mL volumetric flasks. According to the certification of the volumetric flasks, the maximum allowable error for a 100 mL volumetric flask is ± 0.1 mL, and for a 10 mL volumetric flask, it is ± 0.02 mL. (6) Uncertainty of standard curve fitting: The standard uncertainty is expressed as *u* = $\sqrt{{u}_{A}^{2}+{u}_{B}^{2}}$, with the expanded uncertainty formula *U*_*exp*_ = *k* × *u*, where *k* equals the coverage factor (*k* = 2), and the results are detailed in Table 4.

**Table 4:** Estimating measurement uncertainty for 17α-OHP in DBS using ID-LC-MS/MS

Category	Factor	Formula	Level 1	Level 2	Level 3
A	Measurement uncertainty (%)	$\text{S}(\overline{\text{x} })/\sqrt{n}$	3.12	2.67	1.43
B	Calibration curve (%)	$\text{S}(r)/\sqrt{3}$		0.76	
	Purity of CRM (%)	U_CRM_/k_CRM_C		0.10	
	Weighing (%)	$\sqrt{{u}_{m1}^{2}+{u}_{m2}^{2}}$		0.25	
	Pipette (%)	Ecap/$\sqrt{3}$		0.06	
	Volume (%)	$\sqrt{{\text{u}}_{\text{v}1}^{2}+{\text{u}}_{\text{v}2}^{2}}$		0.12	
*U*_*exp*_	*k* = 2	$k\sqrt{{u}_{A}^{2}+{u}_{B}^{2}}$	6.45%	5.58%	3.29%

$\text{S}(\overline{\text{x} })$ is the standard deviation of the measured value, *n* is the number of measurements, $\text{S}(r)$ is the fitted curve’s residual standard deviation, *u*_m1_ is the standard uncertainty introduced by weighing the standard, and *u*_m2_ is the uncertainty introduced by the standard deviation of the balance weighing. The uncertainties in the 100 mL and 10 mL volumetric flasks are v1 and v2, respectively, where Ecap represents the allowable error in the pipette volume, *U*_CRM_ is the expanded uncertainty, *C* is the standard purity, and *k*_CRM_ is the coverage factor.

### Method application

Clinical DBS samples from healthy newborns were categorized by sex, gestational age, and weight (*n* = 214) to determine reference intervals. After statistical analysis, any differences between the groups were examined. If differences were found, the groups were then categorized into reference intervals. The group was based on the determination of preterm birth if the gestational age was less than 37 weeks and a low birth weight if the birth weight was less than 2.5 kg. Statistical analyses were performed to establish the means, standard deviations, and reference ranges.

This study included 214 healthy neonates, including 104 males and 110 females, without epigenetic anomalies, who were negative for CAH, and who were unaffected by interfering medications. Among them, 14 neonates had low birth weights, and 23 were preterm. The normality of the data was confirmed through analysis, revealing an approximately normal distribution. The measurement outcomes were not influenced by sex (*P* = 0.98), whereas significant differences were observed in weight and gestational age, as determined by Student’s test. Anomalies were identified when 17α-OHP levels exceeded the upper limit of the reference range, which was set based on a unilateral 95% confidence interval. The application of this method is summarized in Table 5.

**Table 5:** Reference interval for 17α-OHP in healthy newborns

Category	Number = 214	Reference interval (ng/mL)	*P*
Low weight	14	≤ 12.05	0.002
Normal weight	200	≤ 6.57	
Premature infant	23	≤ 9.89	0.007
Term infant	191	≤ 6.74	

### Statistical analysis

The data were analyzed using SPSS 29.0 (IBM Corp., Armonk, NY, USA), and the means, standard deviations, CVs, and correlation coefficients were calculated. Normality was assessed via the skewness-kurtosis test, with outliers identified using stem-and-leaf plots. Group comparisons were conducted using one-way ANOVA, with *P* < 0.05 indicating statistical significance.

## Discussion

The establishment of an RMP provides standardized operational procedures and data processing techniques for medical diagnostics, which are crucial for ensuring data reliability and comparability. Currently, there are no internationally recognized RMPs for the laboratory determination of 17α-OHP. However, research has been published on a cRMP for the measurement of 17α-OHP in serum, with LC-MS/MS being considered an accurate and reliable technique for establishing reference methods for 17α-OHP [12]. The development of a cRMP for detecting 17α-OHP in DBS is essential for providing clinical laboratory measurement results that are traceable and comparable. This study has successfully developed a cRMP for detecting 17α-OHP in DBS using ID-LC-MS/MS. The demonstrated precision, accuracy, and measurement uncertainty highlight the method suitability for clinical diagnostics, particularly in newborn screening for CAH. A traceable and standardized approach facilitates reliable interlaboratory comparisons of results, which is essential for the standardization of clinical laboratory measurements [17]. This standardization offers a foundation for value assignment in EQA and certification of DBS reference material, thereby promoting QC and assurance measures across laboratories [18]. The cRMP we established simplifies the pretreatment process compared to the conventional MS method by Salt et al. [19], comprehensively validates the performance of the method, and thoroughly assesses the uncertainty.

Optimizing sample preparation, a critical yet challenging phase of the analytical process, has been a focus of this study [20]. Adjustments to the sample preparation protocol, including increasing the extraction solution volume and employing room temperature shaking, have minimized potential interference and improved extraction efficiency. This meticulous optimization ensures that the calibrator closely mimics clinical samples, reducing matrix effects and enhancing method reliability. The decision to use 50 μL of 55% hematocrit whole blood for calibrator preparation was informed by the differential migration of blood cells and serum in filter card collections, which impacts the analysis of analytes predominantly present in the blood serum [21, 22]. Additionally, we found that diluting the sample with water, though commonly recommended, could lead to contamination and operational issues in the mass spectrometer due to hemoglobin dissolution and fiber release [23, 24]. We improved the volume of the extraction solution by making it ten times larger than the sample to be extracted. When concentrated through nitrogen blowing, the use of an excess extraction solution can lead to longer processing times. We used room temperature shaking during extraction because high temperatures make it difficult to control volatilization, leading to significant well-to-well differences.

DBS samples are preferred for newborn screening programs due to their small volume and convenient storage and transport properties [25]. When stored under the right conditions, 17α-OHP can remain stable for more than a year, as confirmed by multiple experiments [26]. We compared the correlation and deviation of the 17α-OHP concentrations between the TRFIA method and the LC-MS/MS method. Notably, the accuracy of the LC-MS/MS method was significantly greater than that of the TRFIA method. This may be due to the high structural similarity of some steroid hormones to 17α-OHP, as found by Jean et al. [27]. Therefore, there may be a cross-antigen in the immunological reaction when TRFIA immunological methods measure 17α-OHP [28]. Han et al.’s research indicated that immunological assays of 17α-OHP in DBS appear to be more susceptible to matrix effects, thus making it unsuitable to use DBS samples from non-patients for evaluating the analytical accuracy of immunological assays [29]. Given the inconsistencies of immunological methods with LC-MS/MS detection, establishing specific biological reference intervals for LC-MS/MS is critical [30]. This study’s establishment of reference intervals represents a significant step toward standardized diagnostic practices. A comparative analysis of 214 clinical DBS samples from healthy newborns revealed notable findings consistent with previous research [31, 32], underscoring the efficacy of the method and the necessity of tailored reference intervals for LC-MS/MS analysis.

LC-MS/MS has revolutionized the field of neonatal screening for CAH by providing a robust, sensitive, and specific method for the quantification of 17α-OHP [33]. This technique’s ability to distinguish 17α-OHP from its structurally similar compounds minimizes the false positives often associated with immunoassays, particularly in premature infants or those with non-CAH illnesses. The use of tandem mass spectrometry coupled with liquid chromatography enhances the analytical specificity, enabling the accurate weight of 17α-OHP levels in the complex matrix of DBS. The continuous development and optimization of LC-MS/MS methodologies promise further improvements in the sensitivity, accuracy, and efficiency of 17α-OHP detection in DBS. Innovations such as the introduction of more stable isotope-labeled and advancements in sample preparation techniques could reduce the variability and enhance the throughput of analyses. Additionally, integrating automated workflows and data management systems could streamline the screening process, enabling high-throughput analysis without compromising analytical quality [34, 35].

Despite its advantages, the widespread adoption of LC-MS/MS for 17α-OHP detection faces several challenges, including the need for specialized equipment and technical expertise, as well as higher operational costs compared to traditional immunoassays. Addressing these challenges through the development of cost-effective instruments, training programs for laboratory personnel, and collaborative efforts to share resources among screening centers can enhance access to this advanced technology.

## Conclusion

The utilization of LC-MS/MS for the detection of 17α-OHP in DBS represents a significant advancement in the field of newborn screening. By offering superior analytical performance, this technique improves the diagnostic accuracy for CAH, contributing to better health outcomes for affected neonates. Moving forward, the ongoing refinement of this technology and its methodologies will undoubtedly continue to push the boundaries of what is possible in newborn screening and beyond.

### Supplementary Information

Below is the link to the electronic supplementary material.

Supplementary file1 (DOCX 18.9 KB)

Supplementary file2 (DOCX 375 KB)

## References

[1] Mallappa A, Merke DP. Management challenges and therapeutic advances in congenital adrenal hyperplasia. Nat Rev Endocrinol. 2022;18(6):337–52. 10.1038/s41574-022-00655-w. 35411073 10.1038/s41574-022-00655-w PMC8999997

[2] Balsamo A, Baronio F, Ortolano R, Menabo S, Baldazzi L, Di Natale V, et al. Congenital adrenal hyperplasias presenting in the newborn and young infant. Front Pediatr. 2020;8: 593315. 10.3389/fped.2020.593315. 33415088 10.3389/fped.2020.593315 PMC7783414

[3] Claahsen-van DGH, Speiser PW, Ahmed SF, Arlt W, Auchus RJ, Falhammar H, et al. Congenital adrenal hyperplasia-current insights in pathophysiology, diagnostics, and management. Endocr Rev. 2022;43(1):91–159. 10.1210/endrev/bnab016. 33961029 10.1210/endrev/bnab016 PMC8755999

[4] Sarafoglou K, Merke DP, Reisch N, Claahsen-van DGH, Falhammar H, Auchus RJ. Interpretation of steroid biomarkers in 21-hydroxylase deficiency and their use in disease management. J Clin Endocr Metab. 2023;108(9):2154–75. 10.1210/clinem/dgad134. 36950738 10.1210/clinem/dgad134 PMC10438890

[5] Yoon YA, Woo S, Kim MS, Kim B, Choi YJ. Establishing 17-hydroxyprogesterone cutoff values for congenital adrenal hyperplasia in preterm, low birth weight, and sick newborns. Exp Clin Endocr Diab. 2023;131(4):216–21. 10.1055/a-2022-8399. 10.1055/a-2022-8399 36854385

[6] Seo JY, Park HD, Kim JW, Oh HJ, Yang JS, Chang YS, et al. Steroid profiling for congenital adrenal hyperplasia by tandem mass spectrometry as a second-tier test reduces follow-up burdens in a tertiary care hospital: a retrospective and prospective evaluation. J Perinat Med. 2014;42(1):121–7. 10.1515/jpm-2013-0154. 23989111 10.1515/jpm-2013-0154

[7] Speiser PW, Arlt W, Auchus RJ, Baskin LS, Conway GS, Merke DP, et al. Congenital adrenal hyperplasia due to steroid 21-hydroxylase deficiency: an Endocrine Society Clinical Practice Guideline. J Clin Endocr Metab. 2018;103(11):4043–88. 10.1210/jc.2018-01865. 30272171 10.1210/jc.2018-01865 PMC6456929

[8] Kao PC, Machacek DA, Magera MJ, Lacey JM, Rinaldo P. Diagnosis of adrenal cortical dysfunction by liquid chromatography-tandem mass spectrometry. Ann Clin Lab Sci. 2001;31(2):199–204. 11337910

[9] Shigematsu Y, Yuasa M, Ishige N, Nakajima H, Tajima G. Development of second-tier liquid chromatography-tandem mass spectrometry analysis for expanded newborn screening in Japan. Int J Neonat Screen. 2021;7(3). 10.3390/ijns7030044. 10.3390/ijns7030044 PMC8293176 34287228

[10] Lai F, Srinivasan S, Wiley V. Evaluation of a two-tier screening pathway for congenital adrenal hyperplasia in the New South Wales Newborn Screening Programme. Int J Neonat Screen. 2020;6(3):63. 10.3390/ijns6030063. 10.3390/ijns6030063 PMC7569785 33117905

[11] Cavarzere P, Camilot M, Palma L, Lauriola S, Gaudino R, Vincenzi M, et al. Twenty years of neonatal screening for congenital adrenal hyperplasia in North-Eastern Italy: role of liquid chromatography-tandem mass spectrometry as a second-tier test. Horm Res Paediat. 2022;95(3):255–63. 10.1159/000524170. 10.1159/000524170 35350013

[12] Zhang Q, Zhang L, Lin H, Cai Z, Yan J, Wang Q, et al. Evaluation of a bracketing calibration-based isotope dilution liquid chromatography-tandem mass spectrometry candidate reference measurement procedure for 17alpha-hydroxyprogesterone in human plasma. Anal Bioanal Chem. 2019;411(27):7095–104. 10.1007/s00216-019-02086-5. 31673753 10.1007/s00216-019-02086-5

[13] Choi R, Park HD, Oh HJ, Lee K, Song J, Lee SY. Dried blood spot multiplexed steroid profiling using liquid chromatography tandem mass spectrometry in Korean neonates. Ann Lab Med. 2019;39(3):263–70. 10.3343/alm.2019.39.3.263. 30623618 10.3343/alm.2019.39.3.263 PMC6340850

[14] Qasrawi DO, Boyd JM, Sadrzadeh S. Measuring steroids from dried blood spots using tandem mass spectrometry to diagnose congenital adrenal hyperplasia. Clin Chim Acta. 2021;520:202–7. 10.1016/j.cca.2021.06.005. 34097883 10.1016/j.cca.2021.06.005

[15] Panteghini M. Redesigning the surveillance of in vitro diagnostic medical devices and of medical laboratory performance by quality control in the traceability era. Clin Chem Lab Med. 2023;61(5):759–68. 10.1515/cclm-2022-1257. 36542481 10.1515/cclm-2022-1257

[16] Braga F, Pasqualetti S, Aloisio E, Panteghini M. The internal quality control in the traceability era. Clin Chem Lab Med. 2020;59(2):291–300. 10.1515/cclm-2020-0371. 32639119 10.1515/cclm-2020-0371

[17] Coverdale J, Harrington CF, Solovyev N. Review: advances in the accuracy and traceability of metalloprotein measurements using isotope dilution inductively coupled plasma mass spectrometry. Crit Rev Anal Chem. 2023:1–18. 10.1080/10408347.2022.2162811. 10.1080/10408347.2022.2162811 36637361

[18] Jones G, Delatour V, Badrick T. Metrological traceability and clinical traceability of laboratory results - the role of commutability in External Quality Assurance. Clin Chem Lab Med. 2022;60(5):669–74. 10.1515/cclm-2022-0038. 35179002 10.1515/cclm-2022-0038

[19] Salter SJ, Cook P, Davies JH, Armston AE. Analysis of 17 alpha-hydroxyprogesterone in bloodspots by liquid chromatography tandem mass spectrometry. Ann Clin Biochem. 2015;52(Pt 1):126–34. 10.1177/0004563214530676. 24842631 10.1177/0004563214530676

[20] Grecso N, Zadori A, Barath A, Galla Z, Racz G, Bereczki C, et al. Comparison of different preparation techniques of dried blood spot quality controls in newborn screening for congenital adrenal hyperplasia. PLoS ONE. 2021;16(5): e252091. 10.1371/journal.pone.0252091. 10.1371/journal.pone.0252091 PMC8136632 34015037

[21] Woo S, Rosli N, Choi S, Kwon HJ, Yoon YA, Ahn S, et al. Development of certified reference material for amino acids in dried blood spots and accuracy assessment of disc sampling. Anal Chem. 2022;94(28):10127–34. 10.1021/acs.analchem.2c01349. 35802862 10.1021/acs.analchem.2c01349 PMC9310008

[22] Ducatez F, Pilon C, Ferey J, Marret S, Bekri S, Tebani A. Evaluation of dried-blood spots and a hematocrit-independent procedure in lysosomal diseases screening using multiplexed tandem mass spectrometry assays. Clin Chim Acta. 2023;542: 117278. 10.1016/j.cca.2023.117278. 36871662 10.1016/j.cca.2023.117278

[23] Topbas M, Canbay E, Sezer E, Canda E, Kalkan US, Coker M, et al. Development, optimization and validation of LC-MS/MS method for the determination of DBS GALT enzyme activity. Anal Biochem. 2023;678: 115284. 10.1016/j.ab.2023.115284. 37572839 10.1016/j.ab.2023.115284

[24] Wang HB, Xiao X, Dai W, Peng R, Le J, Feng YQ, et al. Rapid LC-MS/MS detection of 25-hydroxyvitamin D in dried blood spots. Anal Chim Acta. 2023;1283: 341964. 10.1016/j.aca.2023.341964. 37977788 10.1016/j.aca.2023.341964

[25] Freeman JD, Rosman LM, Ratcliff JD, Strickland PT, Graham DR, Silbergeld EK. State of the science in dried blood spots. Clin Chem. 2018;64(4):656–79. 10.1373/clinchem.2017.275966. 29187355 10.1373/clinchem.2017.275966

[26] Grecso N, Zadori A, Szecsi I, Barath A, Galla Z, Bereczki C, et al. Storage stability of five steroids and in dried blood spots for newborn screening and retrospective diagnosis of congenital adrenal hyperplasia. PLoS ONE. 2020;15(5): e233724. 10.1371/journal.pone.0233724. 10.1371/journal.pone.0233724 PMC7259505 32470014

[27] Fiet J, Le Bouc Y, Guechot J, Helin N, Maubert MA, Farabos D, et al. A liquid chromatography/tandem mass spectometry profile of 16 serum steroids, including 21-deoxycortisol and 21-deoxycorticosterone, for management of congenital adrenal hyperplasia. J Endocr Soc. 2017;1(3):186–201. 10.1210/js.2016-1048. 29264476 10.1210/js.2016-1048 PMC5686660

[28] Al-Lami RA. Immune effects of 17alpha-hydroxyprogesterone caproate. Am J Obstet Gynecol. 2022;227(4):671–5. 10.1016/j.ajog.2022.06.056. 35779586 10.1016/j.ajog.2022.06.056

[29] Han L, Tavakoli NP, Morrissey M, Spink DC, Cao ZT. Liquid chromatography-tandem mass spectrometry analysis of 17-hydroxyprogesterone in dried blood spots revealed matrix effect on immunoassay. Anal Bioanal Chem. 2019;411(2):395–402. 10.1007/s00216-018-1449-0. 10.1007/s00216-018-1449-0 30456606

[30] van der Veen A, van Faassen M, de Jong W, van Beek AP, Dijck-Brouwer D, Kema IP. Development and validation of a LC-MS/MS method for the establishment of reference intervals and biological variation for five plasma steroid hormones. Clin Biochem. 2019;68:15–23. 10.1016/j.clinbiochem.2019.03.013. 30922617 10.1016/j.clinbiochem.2019.03.013

[31] Bae YJ, Zeidler R, Baber R, Vogel M, Wirkner K, Loeffler M, et al. Reference intervals of nine steroid hormones over the life-span analyzed by LC-MS/MS: effect of age, gender, puberty, and oral contraceptives. J Steroid Biochem. 2019;193: 105409. 10.1016/j.jsbmb.2019.105409. 10.1016/j.jsbmb.2019.105409 31201927

[32] de Hora MR, Heather NL, Webster D, Albert BB, Hofman PL. Birth weight- or gestational age-adjusted second-tier LCMSMS cutoffs improve newborn screening for CAH in New Zealand. J Clin Endocr Metab. 2021;106(9):e3390–9. 10.1210/clinem/dgab383. 34058748 10.1210/clinem/dgab383

[33] Gaudl A, Kratzsch J, Ceglarek U. Advancement in steroid hormone analysis by LC-MS/MS in clinical routine diagnostics - a three year recap from serum cortisol to dried blood 17alpha-hydroxyprogesterone. J Steroid Biochem. 2019;192: 105389. 10.1016/j.jsbmb.2019.105389. 10.1016/j.jsbmb.2019.105389 31158444

[34] Lahr RG, Sharma P, Maus A, Langman LJ, Jannetto PJ. Development of an LC-MS/MS assay with automated sample preparation for phosphatidylethanol (PEth)- Not your typical clinical marker. J Chromatogr B. 2023;1229: 123886. 10.1016/j.jchromb.2023.123886. 10.1016/j.jchromb.2023.123886 37714050

[35] Gu Y, Jiang F, Yuan X, Yu F, Liang Y, Xiao C, et al. A novel automated multi-cycle magnetic solid-phase extraction coupled to LC-MS/MS to study the disorders of six functional B vitamins in patients with gastroenterology and hyperhomocysteinemia. J Pharmaceut Biomed. 2024;241: 115989. 10.1016/j.jpba.2024.115989. 10.1016/j.jpba.2024.115989 38271858

