# Supplementary material for: Development and evaluation of a candidate reference measurement procedure for detecting 17α-hydroxyprogesterone in dried blood spots using isotope dilution liquid chromatography tandem mass spectrometry
Source: Anal Bioanal Chem. 2024 Jun 29;416(20):4635–45. doi: 10.1007/s00216-024-05411-9 (PMC11294408; doi:10.1007/s00216-024-05411-9)
Supplement: Supplementary file 1 — Supplementary file1 (DOCX 18.9 KB) [file 216_2024_5411_MOESM1_ESM.docx]

**Table S1.** Optimized binary flow gradient.

| Time(min) | Flow(mL/min) | % B | Cure |
| --- | --- | --- | --- |
| initial | 0.3 | 45 | initial |
| 0.10 | 0.3 | 45 | 6 |
| 2.00 | 0.3 | 80 | 6 |
| 3.00 | 0.3 | 98 | 6 |
| 4.50 | 0.3 | 98 | 6 |
| 4.51 | 0.3 | 45 | 6 |
| 5.00 | 0.3 | 45 | 6 |

**Table S2.** Precision of measuring 17α-OHP in DBS by ID–LC‒MS/MS.

| Level | Concentration  (ng/mL) | Day | Mean  (ng/mL) | Intra-day  (CV,%) | Inter-day  (CV,%) |
| --- | --- | --- | --- | --- | --- |
| 1 | 5.61 | 1 | 5.47 | 5.69 | 5.78 |
|  |  | 2 | 5.40 | 4.87 |  |
|  |  | 3 | 5.25 | 4.08 |  |
|  |  | 4 | 5.25 | 3.73 |  |
|  |  | 5 | 5.57 | 5.21 |  |
| 2 | 13.86 | 1 | 13.90 | 2.71 | 3.48 |
|  |  | 2 | 13.60 | 4.50 |  |
|  |  | 3 | 13.70 | 4.28 |  |
|  |  | 4 | 13.79 | 2.03 |  |
|  |  | 5 | 13.48 | 3.57 |  |
| 3 | 30.36 | 1 | 30.10 | 2.26 | 1.66 |
|  |  | 2 | 30.34 | 2.03 |  |
|  |  | 3 | 29.89 | 1.27 |  |
|  |  | 4 | 29.86 | 2.00 |  |
|  |  | 5 | 30.14 | 2.04 |  |

Table S3. Evaluation of matrix effects of the method.

| Blood spot samples | | Standard solution | |  |
| --- | --- | --- | --- | --- |
| Sample name | Peak area | Sample name | Peak area | matrix effects(%) |
| Sample-L-1 | 69126 | SampleL-1 | 75698 | -8.68 |
| Sample-L-2 | 68783 | SampleL-2 | 75480 | -8.87 |
| Sample-L-3 | 68388 | SampleL-3 | 75305 | -9.18 |
| Sample-L-4 | 68756 | SampleL-4 | 75102 | -8.45 |
| Sample-L-5 | 68394 | SampleL-5 | 75806 | -9.78 |
| Sample-M-1 | 296479 | SampleM-1 | 318186 | -6.82 |
| Sample-M-2 | 290286 | SampleM-2 | 317836 | -8.67 |
| Sample-M-3 | 292576 | SampleM-3 | 317089 | -7.73 |
| Sample-M-4 | 297039 | SampleM-4 | 319535 | -7.04 |
| Sample-M-5 | 296845 | SampleM-5 | 319094 | -6.97 |
| Sample-H-1 | 807364 | SampleH-1 | 866232 | -6.80 |
| Sample-H-2 | 812369 | SampleH-2 | 872604 | -6.90 |
| Sample-H-3 | 807761 | SampleH-3 | 869506 | -7.10 |
| Sample-H-4 | 816831 | SampleH-4 | 873546 | -6.49 |
| Sample-H-5 | 810773 | SampleH-5 | 872971 | -7.12 |

The matrix effect was calculated as$\frac{A_{m-}A_{r}}{A_{r}}$×100%, Am: the area under the curve of a measured substance in a blood spot sample. Ar: the area under the curve of a standard solution. The standard solution is the substance to be measured dissolved in 50% methanol in water. Sample-L-1 represents low-concentration sample 1, and so on; Sample-M-1 represents medium-concentration sample 1; Sample-H-1 represents high-concentration sample 1.

**Table S4.** Examination of the carry-over contamination rate of the method

| Sample name | Measured value(ng/mL) | L-L | H-L |
| --- | --- | --- | --- |
| L1 | 4.86 |  |  |
| L2 | 5.16 | 5.16 |  |
| L3 | 5.56 | 5.56 |  |
| H1 | 30.37 |  |  |
| H2 | 31.55 |  |  |
| L4 | 5.43 |  | 5.43 |
| H3 | 32.66 |  |  |
| H4 | 31.22 |  |  |
| L5 | 4.87 |  | 4.87 |
| L6 | 4.85 | 4.85 |  |
| L7 | 5.16 | 5.16 |  |
| L8 | 4.94 | 4.94 |  |
| H5 | 30.71 |  |  |
| H6 | 30.19 |  |  |
| L9 | 5.00 |  | 5.00 |
| H7 | 31.79 |  |  |
| H8 | 29.36 |  |  |
| L10 | 5.37 |  | 5.37 |
| H9 | 29.94 |  |  |
| H10 | 30.16 |  |  |
| L11 | 5.43 |  | 5.43 |
| Mean |  | 5.14 | 5.22 |
| Carryover (%) |  | - | 1.64 |

The high-concentration sample was diluted ten times to obtain a low-concentration sample. L-L denotes the measured value of the second low-value sample when two consecutive low-value samples are detected. H-L denotes the measured value of the low-value sample after the high-value sample is finished.

**Table S5.** Measurements of method detection limits.

| Sample name | theoretical value(ng/mL) | Measured mean(ng/mL) | S/N | *CV*(%) | Deviation(%) |
| --- | --- | --- | --- | --- | --- |
| Sample1 | 0.06 | 0.10 | 1.74 | 33.53 | 84.62 |
| Sample2 | 0.12 | 0.14 | 6.60 | 12.60 | 8.62 |
| Sample3 | 0.25 | 0.26 | 6.90 | 8.49 | 4.31 |
| Sample4 | 0.50 | 0.52 | 23.35 | 4.73 | 3.00 |
| Sample5 | 1.00 | 1.02 | 124.67 | 1.63 | 2.00 |
