# Supplementary material for: Development and evaluation of a candidate reference measurement procedure for detecting 17α-hydroxyprogesterone in dried blood spots using isotope dilution liquid chromatography tandem mass spectrometry
Source: Anal Bioanal Chem. 2024 Jun 29;416(20):4635–45. doi: 10.1007/s00216-024-05411-9 (PMC11294408; doi:10.1007/s00216-024-05411-9)
Supplement: Supplementary file 2 — Supplementary file2 (DOCX 375 KB) [file 216_2024_5411_MOESM2_ESM.docx]

**Fig. S1** 17α-OHP standard curve. Repeat the test three times per calibrators.


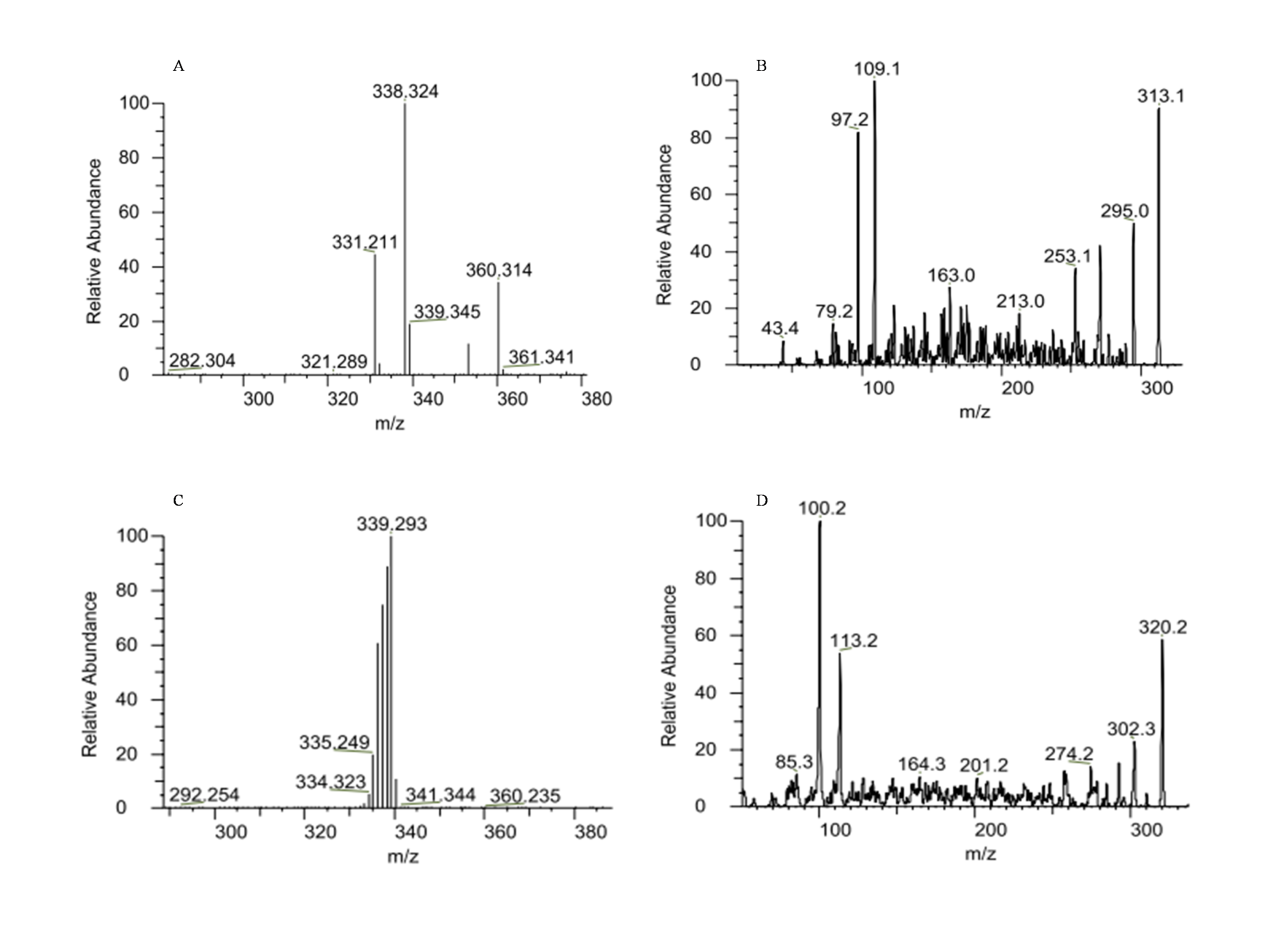


**Fig. S2** Fragmentation ion grams for 17α-OHP and D8-17α-OHP.
